# Predictors of the length of stay in psychiatric inpatient units: a retrospective study for the Paris Psychiatry Hospital Group

**DOI:** 10.3389/fpsyt.2024.1463415

**Published:** 2024-09-18

**Authors:** David Barruel, Anne Perozziello, Hassina Lefèvre, Annie Msellati, Corine Launay, Valérie Dauriac-Le Masson

**Affiliations:** Groupement Hospitalier Universitaire (GHU) Paris Psychiatrie et Neurosciences, Hôpital Sainte Anne, Paris, France

**Keywords:** long stay, patient’s pathway, clinical severity, treatment compliance, treatment resistance, machine learning

## Abstract

**Objective:**

Shortening the length of hospital stay (LOS) has become a major challenge for psychiatric hospitals in reducing unnecessary costs and improving the patient healthcare experience. We investigated the key factors associated with a long psychiatric hospitalization.

**Method:**

This was a retrospective study of 8,870 full-time psychiatric hospital stays (6,216 patients) in the Paris Psychiatry Hospital Group, with a discharge in 2022. We used machine learning tools and univariate and multivariate methods to explore the impact of demographic, pathway-related, and clinical variables on the LOS.

**Results:**

LOS >30 days was associated with age >55 years {odds ratio [OR] =2 [95% confidence interval 1.7–2.3]}, admission from outside the sectorization zone [OR=1.2 (1.1–1.3)], admission via a psychiatric emergency unit [OR, 1.2 (1.1–1.4)], and some clinical severity markers, such as psychotic disorder diagnosis [OR, 1.5 (1.3–1.7)], mandatory care [request of a third party, OR, 2.5 (2.1–2.9); case of imminent danger, OR, 2.3 (1.9–2.7)], the presence of seclusion and mechanical restraint measures (highlighting the positive effect of restraint duration), the somatic comorbidity for female sex [OR, 1.4 (1.2–1.7)], and treatment resistance [OR, 1.4 (1.2–1.6)]. Conversely, LOS ≤30 days was associated with being in a relationship [OR, 0.6 (0.5–0.8)], admission during a travel-related psychiatric episode [OR, 0.5 (0.3–0.6)], and personality and behavior disorders [OR, 0.7 (0.6–0.9)]. We found no significant association for features such as sex and a lack of treatment compliance.

**Conclusion:**

To our knowledge, this is the first recent study to investigate and highlight the impact of factors related to various illness severity markers, medication adherence, and patient journeys on the length of psychiatric hospital stay. A better understanding of long-stay risk factors might be helpful for optimizing the allocation of medical resources and anticipating tailored therapeutic programs.

## 1 Introduction

The cost of inpatient psychiatry in France was estimated at €13.4 billion in 2007, representing 64% of the total cost of mental healthcare (1). Prolonged hospitalization is associated with an increased consumption of health resources and services in addition to a risk of exceeding hospital capacity. Hence, the cost is an important economic burden for our healthcare system. In addition to the impact on healthcare organizations and expenditure, long hospital stays also lead to a loss of quality of life because of social isolation and high dependency (2, 3). Although the results need to be confirmed, a Cochrane review (4) suggested that a short-stay policy for patients with severe mental illness did not result in a “revolving door pattern” of admission or poor or fragmented delivery of care. This study also highlighted that a short stay significantly improved social functioning (including unemployment). Similarly, international recommendations on mental healthcare have promoted the reduction of unnecessary long stay hospitalizations by improving the quality of care (2, 5, 6). In France, this objective has been included in the national plan for the reform of psychiatric financing through a financial incentive indicator (7) based on counting hospital stay days for full-time and free inpatient psychiatric admissions. It aims to encourage psychiatric institutions to minimize inappropriate long stays.

To achieve this goal, we need to better understand the clinical, social, and care pathway determinants that influence the length of psychiatric hospital stay (LOS). Knowledge of the predictors of LOS could help caregivers anticipate this event (8, 9) and thus adapt care planning for earlier discharge (10–12). The benefits are optimized and improve healthcare, reduce the risk of chronic mental illness, and reduce superfluous costs associated with inpatient care.

Reviews of the international literature (8, 10, 13, 14) have identified several factors associated with a risk of a long psychiatric hospital stay: sociodemographic (sex, age, marital status, precariousness, and unemployment), clinical [diagnosis of psychosis, addictive comorbidity, hetero-aggressive behavior, mandatory admission, severity of pathology, suicide risk, and treatment with clozapine or electroconvulsive therapy (ECT)], and organizational (hospital size and number of care coordinators). However, the same patient characteristics may have a different impact on LOS in different countries. Therefore, national studies must take into account the national and contextual complexity of the factors predicting LOS (10). The French literature has highlighted the role of characteristics such as a high level of dependency, diagnosis of psychosis, psychological development disorder, intellectual disability, mandatory admission, seclusion, and mechanical restraint during the stay, as well as territorial health and social care provision (3, 15).

There are few recent studies conducted in France that have investigated factors associated with LOS. The LOS cutoffs used in these studies, 90 (7) and 292 days (3, 15), were very high and do not seem relevant to clinical practice, according to the experience of health professionals. Furthermore, to the best of our knowledge, there is a lack of research in France on the characteristics of the patient journey. French psychiatric care organizations are characterized by a division into sectors, each providing care for approximately 70,000 inhabitants from a determined geographic area. The objective of sector organization was to provide residents from the same geodemographic area some integrated and graduated care, in accordance with the stage of their disease and available as closely as possible to their living space (16, 17). This organization was built to ensure equal access, proximity, continuity, and also multidisciplinarity for inpatient and outpatient care services. The medico-psychological center is the pivotal point of care supply, including prevention, diagnose, ambulatory care, and home-based intervention. In addition to inpatient full-time hospitalization, alternative pathways were developed, such as full-time hospitalization in rehabilitation units, therapeutic apartments, hospitalization at home, and also part-time day or night hospitalization. No previous national study has investigated the impact of admission in a ward outside the patient’s sectorization zone on LOS. Additionally, other pathway features, such as hospitalization during a travel-related psychiatric episode or after a visit to a psychiatric emergency department, have not been investigated. We also did not find any recent French work on the effect of somatic comorbidity, duration of seclusion and physical restraint, medication adherence, and treatment resistance. These characteristics might be associated with a long hospital stay.

The aim of the present exploratory study was to better understand the determinants of long hospital stay outcomes in the French context, using some already established risk factors and exploring new and unstudied candidate features. We used structured and textual data from the electronic health record database of the Paris Psychiatry Hospital Group. This was an opportunity to explore a new approach, combining state-of-the art machine learning tools and statistical methods.

## 2 Methods

### 2.1 Design and data collection

**Figure 1:**
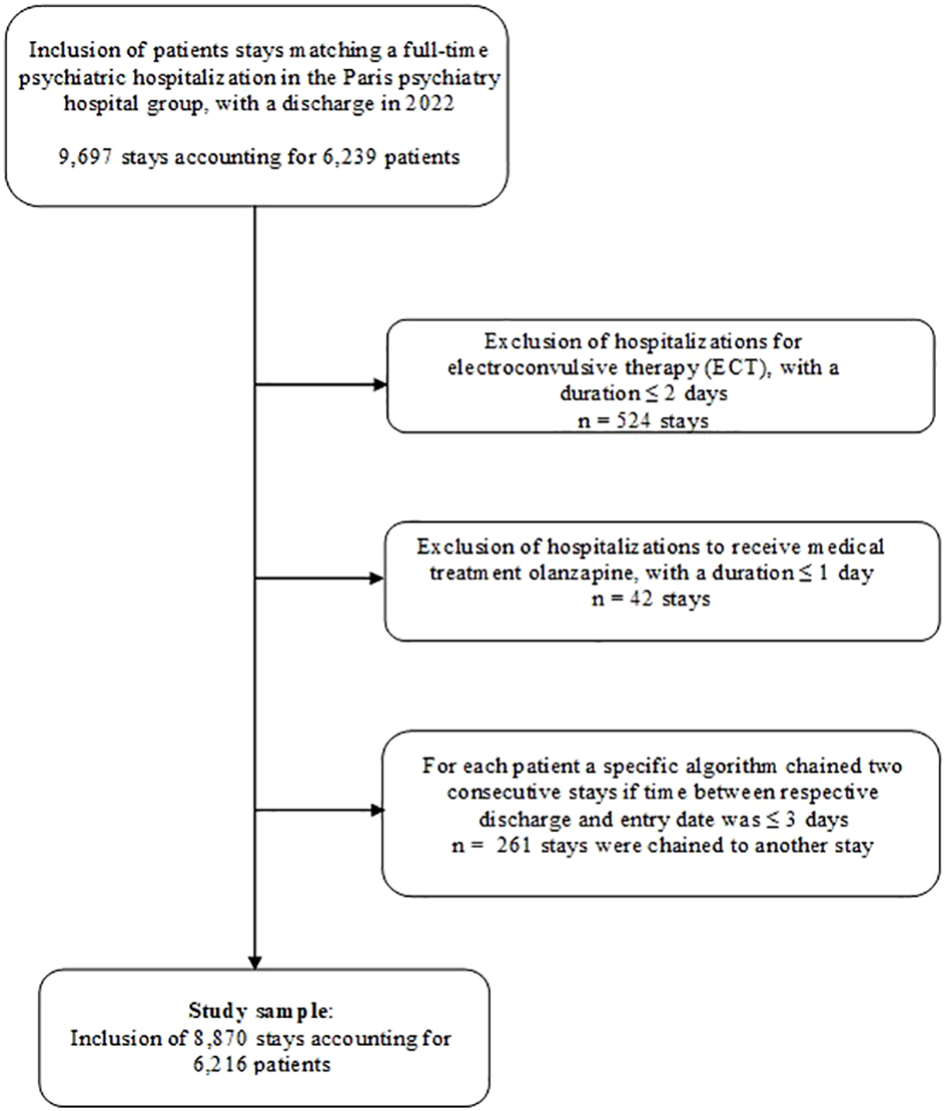
Patient stays selection flowchart.

For this observational study, we used administrative, sociodemographic, and clinical data from the electronic health record database of the Paris Psychiatry Hospital Group, which serves a population of approximately 1.6 million people and covers three-quarters of the city of Paris. Our source database contained 700,000 patient electronic health records. We leveraged structured and unstructured data (e.g., discharge summaries or medical observation reports). We selected patient stays corresponding to a full-time psychiatric hospitalization for patients ≥15 years old, with a discharge in 2022 (n=9,697 admissions). As detailed in **Figure 1**, we excluded short hospitalizations (≤2 days) and iterative hospitalizations when they were dedicated to specific therapies such as ECT in case of treatment-resistant depression or long-acting neuroleptic injection for patients with psychoses. This study was approved by the Paris Psychiatry Hospital Group Research Ethics Committee (accreditation no. 2024-CER-A-007).

#### 2.1.1 Outcome definition

LOS was the study outcome. To assess actual stay duration, we used a specific algorithm: we chained 2 consecutive stays as one stay if the time between the discharge and entry date was ≤3 days. We assumed these stays were the same healthcare period interrupted by a temporary absence. We also treated the LOS outcome as a continuous variable or binary variable by defining “short LOS” as ≤30 days and “long LOS” as >30 days (see **Supplementary Material** for details). This cutoff (30 days) was chosen according to the experience of health professionals. Studies in different contexts also suggest such a threshold (18–20).

#### 2.1.2 Selection of long LOS predictors

The characteristics to be included in the statistical modeling were validated in the literature or hypothesized based on the experience of health professionals. The independent variables were divided into the following categories:

- Demographic variables: sex, age, marital status, social vulnerability (homelessness status, high deprivation index level, and social factors influencing health status).
- Variables characterizing the patient’s care pathway: admission (a) from outside the patient’s sectorization zone, (b) after a travel-related psychiatric episode, and/or (c) after a visit to a psychiatric emergency department.
- Clinical variables: psychiatric primary or associated diagnosis according to the International Classification of Diseases, 10th revision (ICD-10) codes (see **Table 1**), presence of a somatic comorbidity, suicide risk, severity of seclusion and mechanical restraint measure (assessed by the duration of restraint), mandatory admission, number of psychiatric admissions in previous years, medication discontinuation (due to lack of compliance), and treatment resistance.

**Table 1:** International Classification of Diseases, Revision 10 (ICD-10) diagnostic groups.

ICD-10 definition	ICD-10 Code(s)
Organic mental disorders	F00-F09
Mental and behavioral disorders related to alcohol use	F10
Mental and behavioral disorders related to psychoactive substance use except alcohol and tobacco	F11-F19 F17 excluded
Schizophrenia, schizotypal, delusional, and other non-mood psychotic disorders	F20-F29
Bipolar and manic disorders	F30-31
Mood disorders (bipolar and manic episode disorders excluded)	F32-39
Anxiety, dissociative, stress-related, somatoform, and other non-psychotic mental disorders	F40-F49
Behavioral syndromes associated with physiological disturbances and physical factors	F50-F59
Adult personality and behavior disorders	F60-F69
Intellectual disabilities	F70-F79
Pervasive and specific developmental disorders	F80-F89
Behavioral and emotional disorders with onset usually occurring in childhood and adolescence	F90-F99
Social factors influencing health status and contact with health services	Z55-Z65 Z590 (homelessness) excluded

The list of variables with their precise definitions is featured in **Supplementary Material**.

#### 2.1.3 Dataset building

Applying the method mentioned in section 2.1, we obtained a dataset of 8,870 observations, each related to a patient stay. Because of the lack of reliability in the electronic health records for ICD-10 diagnoses related to somatic comorbidity and suicide, we calculated these predictors by combining ICD-10 diagnostic data and information extracted from medical narratives. In the same way, we mined information from textual medical observations to compute the variables medication discontinuation and treatment resistance (for the latter variable, we precisely combined information extracted from narratives with ECT data). For more details about these variables, see **Supplementary Material**. To compute the variables, we applied natural language processing techniques using Python v3.8.5 and the NLTK library. All other predictors were obtained by using only structured information from the electronic health record database, with R v4.3.1 or Python v3.8.5.

### 2.2 Analysis strategy

#### 2.2.1 Descriptive analysis

We performed initial descriptive and univariate analyses of the dataset (n=8,870). Because the distribution of the continuous variables LOS and age was not normal, we first described them using the median [interquartile range (IQR)]. For ease of reading, we also produced mean ± SD. For categorical variables, we described the characteristics of the study population using frequencies and percentages (%).

#### 2.2.2 Bivariate analysis

We first performed preliminary bivariate analysis of the dataset (n=8,870) for each of the 30 selected predictors and the LOS outcome. We transformed the continuous variable age into an ordinal variable for further analysis. We used chi-squared or Fisher’s exact tests for categorical variables and the Cochran–Armitage test for ordinal variables (e.g., age category, seclusion and mechanical restraint severity level, or history of hospitalization in the Paris Psychiatry Hospital Group before 2019; see **Supplementary Material** for definitions). We repeated this bivariate analysis on sex-stratified data (n=8,870).

#### 2.2.3 Multivariate analysis

We then found that a logistic regression with “long LOS” defined as “hospitalization >30 days” was the best model for achieving our study objective. Specifically, we checked whether there was no need for a multilevel model, accounting for a potential nested data structure at the psychiatric sector level (see **Supplementary Material** for more details on model selection). Logistic regression analysis was first used with the dataset for which observations with any missing values were removed (n=6,206). We implemented a model including all the predictors significant at *p*<0.2 on bivariate analysis and interaction terms if necessary (21). Stepwise variable selection was used to select the most relevant variables for prediction. We computed the area under the receiver operating characteristic curve metric (AUC) to evaluate our final multivariate model (see **Supplementary Material** for more details about model evaluation). We estimated odds ratios (ORs) and 95% confidence intervals (CIs), with *p*<0.05 considered statistically significant.

#### 2.2.4 Sensitivity analysis: testing different cutoffs for defining the binary LOS outcome

As a sensitivity analysis, we repeated the whole bivariate and multivariate process, testing different LOS cutoffs for the binary outcome definition, successively 20, 60, and 90 days.

#### 2.2.5 Handling missing data

We applied the same statistical steps to the initial dataset with missing values previously replaced by random imputation (n=8,870) (22). We also checked whether there was a significant difference between stays excluded versus included from the logistic regression process (n=2,664 vs. n=6,206). As a sensitivity analysis, we repeated the same multivariate analysis on a dataset (n=8,522) that filtered the variable mostly affected by missing data (marital status). Statistical analysis was carried out using R v4.3.1.

## 3 Results

### 3.1 Descriptive analysis

**Figure 2:**
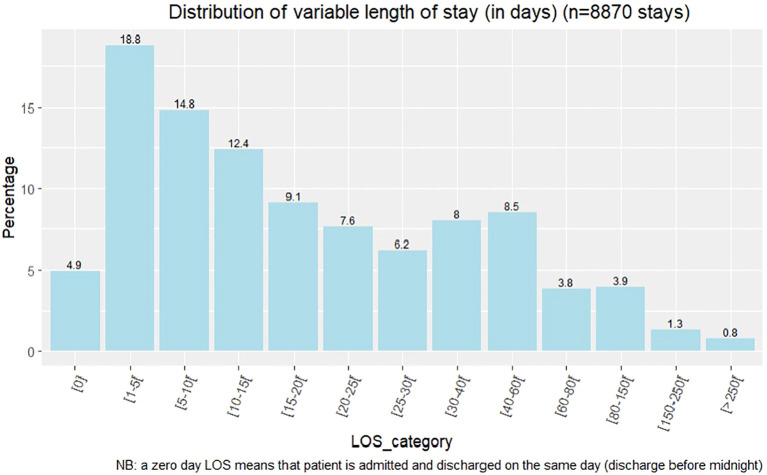
LOS distribution plot.

We constructed a database of 8,870 full-time hospital stays (6,216 patients) (**Figure 1**). The median LOS was 14 days (IQR, 5–31; mean 27 ± 47.8 days) (see distribution plot in **Figure 2**). The median age of patients was 38 (IQR, 27–53; mean age 41 ± 16 years); 44.9% of long stays were for female patients (**Table 2**). Overall, 25.5% of the stays were in a psychiatric ward that was different from the patient’s psychiatric sector of origin, 10.0% were during a travel-related psychiatric episode, and 68.9% were after a visit to a psychiatric emergency unit. We found that 25.1% of the admissions were related to mandatory care at the request of a third party, and 18.7% to imminent danger. In all, 9.1% of stays were associated with another type of mandatory care (by decision of the state representative, of detained persons, after a decision of criminal irresponsibility, or within the framework of a temporary placement order). The two most common diagnoses were schizophrenia and mood disorders, accounting for 52.4% and 40.3% of the 8,870 hospitalizations, respectively. A total of 20.9% of stays featured suicidal risk and 53.5% featured somatic comorbidity.

**Table 2:** Bivariate analysis of patients with a length of psychiatric hospital stay of ≤30 versus >30 days for both sexes and by sex.

		Both sexes			Males			Females		
		≤30	>30		≤30	>30		≤30	>30	
	n=8,870	n=6,613	n=2,257	p-value^1^	n=3,591	n=1,293	p-value^1^	n=3,022	n=964	*p*-value^1^
Demographic variables										
Sex				0.01						
Male	4,884 (55.1)	3,591 (54.3)	1,293 (57.3)		3,591 (100)	1,293 (100)		0 (0)	0 (0)	
Female	3,986 (44.9)	3,022 (45.7)	964 (42.7)		0 (0)	0 (0)		3,022 (100)	964 (100)	
Age category, years				<0.001			0.007			<0.001
≤55	6,928 (78.1)	5,297 (80.1)	1,631 (72.3)		2,979 (83)	1,029 (79.6)		2,318 (76.7)	602 (62.4)	
>55	1,942 (21.9)	1,316 (19.9)	626 (27.7)		612 (17)	264 (20.4)		704 (23.3)	362 (37.6)	
In a relationship (marital status)				<0.001			<0.001			0.1
Yes	881 (9.9)	693 (10.5)	188 (8.3)		295 (8.2)	69 (5.3)		398 (13.2)	119 (12.3)	
NA	2,031 (22.9)	1,593 (24.1)	438 (19.4)		890 (24.8)	273 (21.1)		703 (23.3)	165 (17.1)	
Experiencing homelessness = yes	846 (9.5)	631 (9.5)	215 (9.5)	1	493 (13.7)	155 (12)	0.1	138 (4.6)	60 (6.2)	0.05
High deprivation index level				0.4			0.7			0.2
Yes	4,321 (48.7)	3,207 (48.5)	1,114 (49.4)		1,866 (52.0)	665 (51.4)		1,341 (44.4)	449 (46.6)	
NA	234 (2.6)	172 (2.6)	62 (2.7)		116 (3.2)	40 (3.1)		56 (1.9)	22 (2.3)	
Social factors influencing health status (ICD-10 codes^2^ Z55-Z65) = Yes	824 (9.3)	582 (8.8)	242 (10.7)	0.007	376 (10.5)	160 (12.4)	0.07	206 (6.8)	82 (8.5)	0.09
Variables characterizing the patient’s path										
Admission to a psychiatric ward different from the patient’s psychiatric origin sector = yes	2,260 (25.5)	1,626 (24.6)	634 (28.1)	0.001	889 (24.8)	355 (27.5)	0.06	737 (24.4)	279 (28.9)	0.005
Admission during a travel-related psychiatric episode = yes	886 (10)	744 (11.3)	142 (6.3)	<0.001	487 (13.6)	95 (7.3)	<0.001	257 (8.5)	47 (4.9)	<0.001
Admission after a visit to a psychiatric emergency unit = yes	6,107 (68.9)	4,523 (68.4)	1,584 (70.2)	0.1	2,425 (67.5)	904 (69.9)	0.1	2,098 (69.4)	680 (70.5)	0.5
Clinical variables										
Mandatory care in the request of a third party = yes	2,225 (25.1)	1,432 (21.7)	793 (35.1)	<0.001	789 (22)	440 (34)	<0.001	643 (21.3)	353 (36.6)	<0.001
Mandatory care in case of imminent danger = yes	1,656 (18.7)	1,137 (17.2)	519 (23)	<0.001	632 (17.6)	266 (20.6)	0.02	505 (16.7)	253 (26.2)	<0.001
Other type of mandatory care^3^ = yes	810 (9.1)	418 (6.3)	392 (17.4)	<0.001	367 (10.2)	314 (24.3)	<0.001	51 (1.7)	78 (8.1)	<0.001
Organic mental disorders (ICD-10 codes F00-F09)				<0.001			0.2			<0.001
Yes	175 (2)	105 (1.6)	70 (3.1)		61 (1.7)	30 (2.3)		44 (1.5)	40 (4.1)	
NA^4^	348 (3.9)	280 (4.2)	68 (3)		149 (4.1)	28 (2.2)		131 (4.3)	40 (4.1)	
Mental and behavioral disorders related to alcohol use (ICD-10 code F10)^4^ = yes	1,166 (13.1)	908 (13.7)	258 (11.4)	0.003	567 (15.8)	184 (14.2)	0.1	341 (11.3)	74 (7.7)	0.001
Mental and behavioral disorders related to psychoactive substance use except alcohol and tobacco (ICD-10 codes F11-F12-F13-F14-F15-F16-F18-F19)^4^ = yes	1,656 (18.7)	1,228 (18.6)	428 (19)	0.9	862 (24)	328 (25.4)	0.5	366 (12.1)	100 (10.4)	0.1
Schizophrenia and other psychotic disorders (ICD-10 codes F20-F29)^4^ = yes	4,644 (52.4)	3,118 (47.1)	1,526 (67.6)	<0.001	1974 (55)	938 (72.5)	<0.001	1,144 (37.9)	588 (61)	<0.001
Bipolar and manic disorders (ICD-10 codes F30-F31)^4^ = yes	1,423 (16)	1,050 (15.9)	373 (16.5)	0.7	453 (12.6)	160 (12.4)	0.7	597 (19.8)	213 (22.1)	0.1
Mood disorders (bipolar and manic episode disorders excluded) (ICD-10 codes F32-39)^4^ = yes	2,479 (27.9)	2,074 (31.4)	405 (17.9)	<0.001	922 (25.7)	171 (13.2)	<0.001	1,152 (38.1)	234 (24.3)	<0.001
Anxiety disorders (ICD-10 codes F40-F49)^4^ = yes	1,299 (14.6)	1,095 (16.6)	204 (9)	<0.001	478 (13.3)	108 (8.4)	<0.001	617 (20.4)	96 (10)	<0.001
Behavioral syndromes associated with physiological disturbances and physical factors (ICD-10 codes F50-F59)^4^ = yes	205 (2.3)	167 (2.5)	38 (1.7)	0.02	25 (0.7)	12 (0.9)	0.5	142 (4.7)	26 (2.7)	0.008
Adult personality and behavior disorders (ICD-10 codes F60-F69)^4^ = yes	1,483 (16.7)	1,235 (18.7)	248 (11)	<0.001	546 (15.2)	126 (9.7)	<0.001	689 (22.8)	122 (12.7)	<0.001
Intellectual disabilities (F70-F79)^4^ = yes	109 (1.2)	85 (1.3)	24 (1.1)	0.4	60 (1.7)	21 (1.6)	0.9	25 (0.8)	3 (0.3)	0.1
Pervasive and specific developmental disorders (ICD-10 codes F80-F89)^4^ = yes	272 (3.1)	222 (3.4)	50 (2.2)	0.006	165 (4.6)	35 (2.7)	0.003	57 (1.9)	15 (1.6)	0.6
Behavioral and emotional disorders with onset usually occurring in childhood and adolescence (ICD-10 codes F90-F99)^4^ = yes	176 (2)	144 (2.2)	32 (1.4)	0.03	89 (2.5)	22 (1.7)	0.1	55 (1.8)	10 (1)	0.1
Suicide risk = yes	1,850 (20.9)	1,524 (23.0)	326 (14.4)	<0.001	709 (19.7)	162 (12.5)	<0.001	815 (27.0)	164 (17.0)	<0.001
Somatic comorbidity = yes	4,746 (53.5)	3,297 (49.9)	1449 (64.2)	<0.001	1,784 (49.7)	776 (60)	<0.001	1,513 (50.1)	673 (69.8)	<0.001
Seclusion and mechanical restraint severity level				<0.001			<0.001			<0.001
No restraint provided during the stay	7,342 (82.8)	5,863 (88.7)	1,479 (65.5)		3,079 (85.7)	757 (58.5)		2,784 (92.1)	722 (74.9)	
A restraint provided during the stay with (a) seclusion restraint <168 h and (b) physical restraint <48 h	710 (8)	462 (7)	248 (11)		296 (8.2)	159 (12.3)		166 (5.5)	89 (9.2)	
A therapeutic restraint provided during the stay with (a) seclusion restraint ≥168 h or (b) physical restraint ≥48 h	818 (9.2)	288 (4.4)	530 (23.5)		216 (6)	377 (29.2)		72 (2.4)	153 (15.9)	
Hospitalization history severity level				<0.001			<0.001			<0.001
No hospitalization before 2019	5,349 (60.3)	4,195 (63.4)	1,154 (51.1)		2,214 (61.7)	677 (52.4)		1,981 (65.6)	477 (49.5)	
Number of hospitalizations before 2019 in the Paris Psychiatry Hospital Group >1 and ≤3	1,741 (19.6)	1,225 (18.5)	516 (22.9)		681 (19)	290 (22.4)		544 (18)	226 (23.4)	
Number of hospitalizations before 2019 in the Paris Psychiatry Hospital Group >3	1,780 (20.1)	1,193 (18)	587 (26)		696 (19.4)	326 (25.2)		497 (16.4)	261 (27.1)	
Treatment discontinuation (due to lack of compliance) = yes	2,237 (25.2)	1,537 (23.2)	700 (31)	<0.001	848 (23.6)	396 (30.6)	<0.001	689 (22.8)	304 (31.5)	<0.001
Treatment resistance = yes	2,339 (26.4)	1,534 (23.2)	805 (35.7)	<0.001	840 (23.4)	469 (36.3)	<0.001	694 (23)	336 (34.9)	<0.001

^1^p was calculated on data with observations with missing values removed.

^2^ICD-10: International Classification of Diseases, 10th revision.

^3^Mandatory care (a) by decision of the state representative or (b) of detained persons or (c) following a decision of criminal irresponsibility or (d) within the framework of a temporary placement order.

^4^NA: descriptive statistical analysis of missing values is the same for the following ICD-10 diagnostic codes: F0, F10, F11-19, F2, F30-31, F32-39, F4, F5, F6, F7, F8, and F9.

### 3.2 Bivariate and multivariate analysis

#### 3.2.1 Bivariate analysis of patients with a length of psychiatric hospital stay ≤30 versus >30 days

**Table 2** reports details of the results by sex and overall. A long LOS was significantly increased for male patients (*p*=0.01) and aged >55 years (*p*<0.001), persons not in a relationship (*p*<0.001), and a diagnosis related to social factors influencing health status (*p*=0.007). Long versus short LOS was less frequent with travel-related psychiatric episodes (*p*<0.001) but was more frequent for patients admitted outside their sectorization zone (*p*=0.001). Long versus short LOS was more frequent for patients under mandatory care (*p*<0.001 for all mandatory care predictors). The clinical diagnostic profile of patients significantly differed between the patients with long and short LOS: long LOS was associated with a diagnosis of schizophrenia (*p*<0.001), organic mental disorders (*p*<0.001), or a somatic comorbidity (*p*<0.001). In addition, long LOS was associated with seclusion and mechanical restraint severity level, hospitalization history severity level, medication discontinuation (because of a lack of compliance), and treatment resistance (*p*<0.001 for four factors). However, a long LOS was less frequent among patients with disorders related to alcohol use (*p*=0.003), mood disorders (excluding bipolar and manic episodes) (*p*<0.001), anxiety (*p*<0.001), behavioral syndromes associated with physiological disturbances (*p*=0.02), personality disorders (*p*<0.001), psychological developmental disorders (*p*=0.006), behavioral and emotional disorders with early onset (*p*=0.03), and suicide risk (*p*<0.001).

#### 3.2.2 Multivariate logistic regression analysis of factors associated with the odds of a psychiatric hospital stay of >30 days

The odds of a long LOS was associated with age >55 years [OR, 2 (95% CI 1.7–2.3)], admission from outside the sectorization zone [OR, 1.2 (1.1–1.3)] and admission via a psychiatric emergency unit [OR, 1.2 (1.1–1.4)], mandatory care [request of a third party [OR, 2.5 (2.1–2.9)], in imminent danger [OR, 2.3 (1.9–2.7)], another type of mandatory care [OR, 4.3 (3.5–5.5)]], a diagnosis of schizophrenia [OR, 1.5 (1.3–1.7)], a high level of seclusion and mechanical restraint [level 1: OR, 1.6 (1.3–1.9); level 2: OR, 5 (4–6.2)], and treatment resistance [OR, 1.4 (1.2–1.6)] (**Table 3**). We found a significant interaction between sex and somatic comorbidity (*p*<0.002): a sex-stratified analysis confirmed that somatic comorbidity increased the odds of LOS for only females [OR, 1.4 (1.2–1.7), computed after taking interaction terms into account]. Conversely, protective factors for the odds of a long LOS were being in a relationship [OR, 0.6 (0.5-0.8)], admission during a travel-related psychiatric episode [OR, 0.5 (0.3–0.6)], and a diagnosis of personality disorders [OR, 0.7 (0.6,0.9)].

**Table 3:** Multivariate logistic regression analysis of factors associated with the odds of a psychiatric hospital stay of >30 days.

		OR*^1^*	95% CI*^2^*	*p*-value
Demographic variables				
Sex (ref =male)	Female	0.9	0.8-1.2	0.6
Age category, years (ref = ≤ 55)				
Level 1	>55	2	1.7-2.3	<0.001
In a relationship (ref = no)	Yes	0.6	0.5-0.8	<0.001
Variables characterizing the patient’s path				
Admission to a psychiatric ward outside the sectorization zone (ref = no)	Yes	1.2	1.1-1.3	0.04
Admission during a travel-related psychiatric episode (ref=no)	Yes	0.5	0.3-0.6	<0.001
Admission after a visit to a psychiatric emergency unit (ref=no)	Yes	1.2	1.1-1.4	0.003
Clinical variables				
Mandatory care in the request of a third party (ref=no)	Yes	2.5	2.1-2.9	<0.001
Mandatory care in the case of imminent danger (ref=no)	Yes	2.3	1.9-2.7	<0.001
Other type of mandatory care^4^ (ref=no)	Yes	4.3	3.5-5.5	<0.001
Organic mental disorders ICD-10^3^ codes F00-F09 (ref=no)	Yes	1.4	1-2.1	0.07
Schizophrenia and other psychotic disorders ICD-10 codes F20- F29 (ref=no)	Yes	1.5	1.3-1.7	<0.001
Anxiety disorders ICD-10 codes F40-F49 (ref=no)	Yes	0.8	0.7-1	0.06
Adult personality and behavior disorders ICD-10 codes F60- F69 (ref=no)	Yes	0.7	0.6-0.9	0.002
Suicide risk (ref=no)	Yes	0.9	0.7-1	0.09
Somatic comorbidities (ref=no)	Yes	1.2	1-1.4	0.09
Seclusion and mechanical restraint severity level (ref=no restraint was provided during the stay) Restraint measure was provided during the stay according to the following durations for seclusion and physical restraint:				
Level 1	(a) Seclusion restraint <168 h and (b) physical restraint <48 h	1.6	1.3-1.9	<0.001
Level 2	(a) Seclusion restraint ≥168 h or (b) physical restraint ≥48 h	5	4-6.2	<0.001
Treatment resistance (ref = no)	Yes	1.4	1.2-1.6	<0.001
Interaction term: sex * diagnosis of somatic comorbidities	Yes * Yes	1.5	1.2-1.9	0.002

^1^OR = odds ratio.

^2^CI = confidence interval.

^3^ICD-10, International Classification of Diseases, 10th revision.

^4^Other type of mandatory care: (a) by decision of the state representative or (b) of detained persons or (c) after a decision of criminal irresponsibility or (d) within the framework of a temporary placement order.

For receiver operating characteristic (ROC) analysis, the AUC for our final model was 65.9% (95% CI 56.3–74.9). We checked that we had acceptable results for the following tests: variance inflation factor, deviance, and Hosmer and Lemeshow (**Supplementary Material**).

#### 3.2.3 Sensitivity analysis of different cutoff values for binary LOS

Sensitivity analysis consisted of using different cutoff thresholds for LOS, successively 20, 60, and 90 days. This exploration showed consistent results for the final logistic regression. For the three mentioned cutoffs, the diagnosis of organic mental disorders was positively associated with a long stay. Additionally, for homelessness status, for a cutoff of 90 days, the OR was 1.9 (95% CI 1.3–2.7) (**Supplementary Material**).

#### 3.2.4 Impact of missing data handling

We found consistent conclusions for the highlighted variables of interest when we performed bivariate and multivariate analyses, whether imputing or excluding missing data. On the imputed dataset, the organic mental disorders diagnosis appeared as a long-stay risk factor [OR, 1.6 (95% CI 1.1–2.2)]. Nevertheless, observations excluded because of missing values (n=2,664) were significantly associated with male sex, older age, and a long LOS as compared with observations with no missing information (n=6,206) (**Supplementary Material**).

## 4 Discussion

### 4.1 Impact on clinical practice

Factors that increased the odds of a long LOS were age >55 years, admission from outside the patient’s sectorization zone, admission via a psychiatric emergency unit, mandatory care, seclusion and physical restraint, diagnosis of schizophrenia and psychosis, treatment resistance, and somatic comorbidity for female sex. Factors that reduced the odds of a long LOS were being in a relationship, admission during a travel-related psychiatric episode, and a diagnosis of a personality disorder.

Our results agree with the French and international literature. Among patient characteristics previously validated as risk factors, we first identified age >55 years. We found a positive association between age and a long LOS in previous studies (13, 23, 24). The positive association of outcome with a diagnosis of schizophrenia has been widely confirmed (3, 9, 13, 14, 19, 23, 25–28). We also highlighted features related to clinical severity, reported in previous studies: treatment resistance (29, 30), mandatory care (3), and seclusion and mechanical restraint (3) (25). We noted some previous contradictory findings (31) on the effect of coercive treatment on LOS (alternatively positive, negative, or null, depending on the study). However, these results were obtained in different foreign countries with their own mandatory care regulations, but our conclusions were consistent with a recent French study (3). We found another significant result for the severity of seclusion and mechanical restraint: a multiplication factor of 3 for the risk of LOS between the first and second variable level, depending on the duration of the measure. These findings suggest that the use of mandatory care and seclusion and mechanical restraint may correspond to severe and difficult-to-treat symptoms, materialized by patient aggressiveness, dangerousness, a lack of illness awareness, social withdrawal, functional impairment, and a low adherence to care. This observation could explain the need for complex treatment, difficulties in discharge, and thus a long stay (3, 9, 32). The literature suggests that particular attention should be paid to the severity of illness (13).

Another notable finding of our study was the positive association of somatic comorbidity with a long LOS for only women. Somatic illness has been described in the literature as a potential “focus of care” (33) and a risk factor for long hospital stays (13, 34, 35). However, we could not find other examples of this interaction between sex and somatic comorbidity in the literature. To interpret our result, because women are more likely to report physical symptoms than men (36), somatic symptoms may be easier to treat for women within a psychiatry unit, in contrast to men, who have to be transferred to the general medical ward and are therefore discharged from psychiatry care earlier.

Multivariate analysis of imputed data revealed a diagnosis of organic mental disorders as a risk factor for a long LOS. Considering the specific low prevalence of positive cases for this feature, with more statistical power, this characteristic might have initially appeared among the significant predictors. This result was also confirmed with sensitivity analysis with cutoffs of 20, 60, and 90 days. In addition, homelessness became positively and significantly associated with the highest cutoff of LOS (90 days). This finding agreed with previous research reporting the impact of homelessness on LOS (21, 37).

We found a protective effect of non-single marital status on a long LOS, in line with the literature describing it as a proxy “for functional impairment” (13) and a factor “reflecting resilience” (18). This characteristic is related to human relationships and family connection providing moral support to the patient and is consistent with studies showing that family visits reduce the LOS (38). We found no association between the diagnosis of mood disorders and a long stay in our multivariate analysis. Yet, significance would have been in accordance with previous French studies (3) but not the international literature (8, 29, 37). A diagnosis of a personality disorder was a negative predictor, reported in previous research (21, 28, 39). This observation was consistent with international guidelines (40), recommending limited extended hospitalization in this case. Moreover, it was also consistent with the fact that personality disorders are more often a comorbidity rather than a primary cause of full-time hospitalization (for 2019–2022, the unpublished GHU Paris Psychiatry Hospital annual report highlights that 58% of personality disorder diagnoses related to full-time hospitalization in the Paris Psychiatry Hospital Group were “associated” and not “primary” diagnoses).

An interesting result of our exploration was the variables related to the patient pathway. We highlighted two new significant features. The first was admission after a visit via a psychiatry emergency unit: its positive effect on a long LOS could be interpreted as a marker of the clinical severity of the disease and need for more complex care. The second, the protective effect of a long LOS during a travel-related psychiatric episode, was in line with the profile of these patients (41). Such patients are mostly traveling from France (Paris suburbs or other regions) (41). They are out of their sectorization zone, psychotic, and characterized by a delusional state or disorganization of thought. Their vulnerability and lack of control justifies rapid hospitalization to protect themselves and others, but as soon as possible, health professionals organize their return to their original psychiatric sector to ensure better follow-up. In addition, we found admission from outside the sectorization zone positively associated with a long LOS. Sensitivity analysis with a lower cutoff point for LOS (20 days) confirmed this result. This finding was consistent with those from previous studies in the UK (28, 42). Out-of-area patients may be mostly new and unknown patients for psychiatric wards and thus require a long stay. In addition, out-of-area placements might penalize continuity of care and thus increase the LOS (28).

Our current results suggest that these characteristics related to the patient and the journey should be systematically assessed by healthcare professionals and taken into account during clinical decision-making. Knowledge of such determinants of LOS could help clinicians identify and focus on patients with high clinical severity and a risk of a prolonged stay. We suggest that long-stay patients require a more complex behavioral management and thus a higher level of staff in dedicated units, for specialized acute care. The aim would be to provide them tailored care with a multidisciplinary team, to optimize treatment, discharge planning, and use intensive case management for patients with disruptive behaviors (43, 44). Our results also suggested the benefits of early treatment resistance detection (45) and optimization of somatic comorbidity care. Moreover, the protective effect of non-single marital status on a long LOS emphasized the importance for health care professionals to favor any opportunity for patients to have constructive interactions with families, and also with the social environment. This confirmed the benefits of an approach integrating mental health and social care, through rehabilitation programs, assertive community treatment, and care provided in community-based settings (43).

Our findings should also be contextualized in regard to the variety of international results on the same subject. The mean LOS in this study (27 days) approximates the mean LOS highlighted in the international literature for the USA (24.9 days) (13) and Quebec (25.3 days) (43). It is above the mean LOS found for Italy (17.9 days) (10) and India (5.7 days) (46), and below the mean LOS found for other European countries (10) such as Germany (37 days), Poland (33.4 days), the United Kingdom (46.2 days), and Belgium (55.1 days). Previous studies conducted for Germany, Poland, the UK, Italy, and Belgium (10) highlighted features such as poor social functioning, psychotic disorder, and high clinical severity (in line with our findings) that predicted LOS regardless of the country. However, this study argued that the specific effect of variables might be different from one country to another, and that national context characteristics (cultural and organizational) might influence LOS and its determinants. A previous study comparing the USA and Germany (47) suggested that a greater number of social workers and private psychiatrists per inhabitant in the USA might ease a faster connection to after care, and thus partly explain a lower mean LOS in the USA. We suggest that French sectorization might also influence our LOS in the same way: our system provides specific pathways such as alternatives to full-time hospitalization (23) and rehabilitation programs (improving continuity of care, patient autonomy, and reintegration into the community) that might lower our mean LOS, compared with other European countries. A perspective of future studies would be to extend that kind of comparative study (10) to a greater number of countries, including France, with a rigorous approach that takes national healthcare system characteristics into account.

### 4.2 Strengths

Although many studies have explored factors relating to LOS, there are few up-to-date studies conducted in France. We confirmed and broadened results from French studies related to LOS (3, 15, 23, 25). This study had several methodological assets. Our sample size (n=8,870) aligned with literature recommendations (at least 3,000 observations) (13), which ensured that our study was not underpowered, taking the number of covariables we used for modeling into account (48). We used data from the Paris Psychiatry Hospital Group electronic health records. This healthcare institution, consisting of three different hospitals, provides mental healthcare for much of the city of Paris, offering a representative target population of approximately 1.6 million people. Therefore, our sample was characterized by different types of patients with very different sociodemographic features. It was a representative sample of the Paris population and allowed us to minimize selection bias. To our knowledge, this study is the first to compute true LOS with a specific algorithm. With our computation method, we avoided a bias while evaluating LOS: we took into account the potential splitting of the healthcare period with a spurious administrative discharge, normally supposed to be a temporary absence. We performed a comprehensive analysis by including several categories of predictors (demographic, clinical, and related to care pathway) in our final model, selected after several rigorous statistical steps. As far as we know, this research was among the first in France to analyze the effect of factors related to the patient’s pathway, which are important understudied characteristics. We compensated for the lack of structured data for somatic comorbidity and suicide risk by implementing natural language processing methods on raw medical narratives and leveraged comprehensive lexical dictionaries assessed by different raters. We checked that ignoring sector effects did not bias our results significantly (see **Supplementary Material**). Our sensitivity multivariate analysis results were consistent in terms of age, psychotic diagnosis, and clinical severity factors, whatever the cutoff used to define a long LOS. A bivariate comparison of included versus excluded observations revealed the latter observations were significantly associated with older male patients with a long LOS. This finding suggested that removing observations with missing values (necessary before logistic regression implementation) could lead to a potential bias selection, resulting in a minimization of the effect size of predictors in logistic regression modeling. However, we ran consistent sensitivity multivariate analyses on imputed data (n=8,870) and the dataset excluding variable marital status, accountable for most of the missing information (n=8,522). The missing data exclusion did not significantly bias our main findings in multivariate models. These methodological steps strengthened the robustness of our final conclusions.

### 4.3 Limitations and perspectives

Our results should be interpreted in light of several methodological limitations. The first are those related to observational and retrospective studies (49, 50). We could not assess any causal relationship between explanatory variables and long-stay outcome. Our results were at the association level only. Future research on a national level will be needed to ensure better generalizability and address these questions about sector-level LOS determinants more accurately. For instance, it would be relevant to take hospital and ward policies about coercive measures into account, specifically when we investigate the impact of seclusion and restraint on length of stay. There is important literature about programs for reducing seclusion and restraint (51, 52). De-escalation techniques for psychosis-induced aggression or agitation (53), crisis prevention plans (54), tools like advanced directives in psychiatry (55), the use of “sensory rooms” (55), and a focus on a secure therapeutic relationship (56, 57) can help limit tension and the frequency of conflict. It would be interesting in a future study to investigate how these hospital-level and ward-level strategies might contribute to reducing length of stay.

Another limitation was the lack of data availability for some patient characteristics. We were not able to include, among explanatory variables, unemployment, a “proxy for current functional impairment” (21, 58), dependency (3, 25), symptom measurements, which could be used to assess clinical severity more accurately (9, 18, 28, 31), and biochemical markers obtained at the beginning of hospitalization (18). Nor could we evaluate the effect of the treatments received. To deal with polypharmacy impact, suggested strategies might be to stratify patients starting a treatment at the point of inclusion in the study and those already receiving treatment (59), grouping patients depending on the kind of treatment they receive (18). For future research, the medical record could include a traceability of precise reasons preventing patient discharge, whether clinical or organizational (e.g., no place available in a medico-social institution, a lack of rehabilitative programs at discharge, and no possibility of rehousing) (3). Future studies need to include all these factors. Our exploration was a proof of concept for other studies based on extended and national-level data.

## 5 Conclusions

LOS is an increasing focus for psychiatric hospitals, related to the need to reduce unnecessary healthcare costs. In this study, we used statistical and innovative machine learning tools to explore factors related to a long hospitalization. To the best of our knowledge, our study is the first in recent French literature to define a long stay with a clinically relevant duration threshold and focus on under-investigated variables such as the patient’s path characteristics, a somatic comorbidity, seclusion and mechanical restraint duration, and treatment compliance and resistance. Statistical modeling underlined the factors age >55 years, out-of-area hospitalization, and features relevant to illness severity as related to a long LOS. Our results also revealed the protective effect of variables such as admission during a travel-related psychiatric episode, being in a relationship, and a diagnosis of a personality disorder. The risk factors we highlighted should alert and encourage healthcare professionals to anticipate tailored coordinated action for early discharge. Our results are encouraging but we are cautious to generalize them to the French population. To achieve this, supplementary investigation should be carried out on a national scale. Such a study would also better highlight the role of regional characteristics such as healthcare organizations, social care, and housing policies. Future studies should also take factors related to medication patterns and biological analysis results into account. The use of symptom assessment data would also be justified to better reflect illness severity and its evolution during the stay.

## Data Availability

The raw data supporting the conclusions of this article will be made available by the authors, without undue reservation.
