# Supplementary material for: Predictors of the length of stay in psychiatric inpatient units: a retrospective study for the Paris Psychiatry Hospital Group
Source: Front Psychiatry. 2024 Sep 18;15:1463415. doi: 10.3389/fpsyt.2024.1463415 (PMC11445158; doi:10.3389/fpsyt.2024.1463415)
Supplement: Supplementary file 1 [file Table1.docx]

| **SUPPLEMENTARY MATERIAL** |
| --- |

1. **Description of predictors**

| **Feature** | | **Value** | **Definition** | |
| --- | --- | --- | --- | --- |
| **Sociodemographic variables** | | | | |
| Female sex | | yes /no | yes = patient was female | |
| Age category, years  Ordinal variable | | Ref= ≤55  >55 | Age was computed in years at day of admission | |
| In a relationship (marital status) | | yes/no |  | |
| Homeless | | yes/no | yes = (a) an ICD-10^1^ diagnosis code Z590 was recorded during the stay or (b) homelessness status was recorded in specific administrative form | |
| High deprivation index level | | yes/no | For each stay, we defined the French Deprivation Index^2^ (FDEP) according patient place of residence area. We computed the median FDEP value for dataset. We used this value to code the predictor “High deprivation index level” as “yes” if the value for the stay was greater than the “median value” versus “no” in the opposite case | |
| Social factors influencing health status | | yes/no | yes = an ICD-10^1^ diagnosis code from the Z55-Z65 class was recorded between 2019 and 2022, excluding Z590 (homelessness) | |
| **Variables related to care pathway during the stay** | | | | |
| Out of area admission = admission in a psychiatric ward different from the patient’s psychiatric origin sector | | yes/no | This information is normally recorded in a specific administrative form, for which we observed lack of traceability. In case of missing information, we computed this predictor by using a dedicated algorithm examining patient history and place of residence. | |
| Admission during a travel-related psychiatric episode | | yes/no | This information is normally recorded in a specific administrative form, for which we observed lack of traceability. In case of missing information, we computed this predictor by using a dedicated algorithm examining patient history and place of residence. | |
| Admission after a visit in a psychiatric emergency unit | | yes/no | yes = mandatory care was provided during patient stay | |
| **Clinical variables** | | | | |
| Mandatory care in the request of a third party | | yes/no |  | |
| Mandatory care in case of imminent danger | | yes/no |  | |
| Other type of mandatory care | | yes/no |  | |
| (a) by decision of the state representative or  (b) of detained persons or  (c) after a decision of criminal irresponsibility or (d) within the framework of a temporary placement order | |  |  | |
| Main diagnosis | |  | yes = an ICD-10^1^ diagnosis code from the following classes was recorded between 2019 and 2022 | |
| Organic mental disorders | yes/no | | | F00-F09 |
| Mental and behavioral disorders related to alcohol use | yes/no | | | F10 |
| Mental and behavioral disorders related to psychoactive substance use except alcohol and tobacco | yes/no | | | F11-F19 (F17 excluded) |
| Schizophrenia, schizotypal, delusional, and other non-mood psychotic disorders | yes/no | | | F20-F29 |
| Bipolar and manic disorders | yes/no | | | F30-31 |
| Mood disorders (bipolar and manic episode disorders excluded) | yes/no | | | F32-39 |
| Anxiety, dissociative, stress-related, somatoform and other nonpsychotic mental disorders | yes/no | | | F40-F49 |
| Behavioral syndromes associated with physiological disturbances and physical factors | yes/no | | | F50-F59 |
| Adult personality and behavior disorders | yes/no | | | F60-F69 |
| Intellectual disabilities | yes/no | | | F70-F79 |
| Pervasive and specific developmental disorders | yes/no | | | F80-F89 |
| Behavioral and emotional disorders with onset usually occurring in childhood and adolescence | yes/no | | | F90-F99 |
| Suicide risk | | yes/no | yes = (a) an ICD-10^1^ diagnosis code from the Z915/R458/X60-X84 class was recorded between 2019 and 2022 or (b) a positive suicide risk assessment was text-mined^3^ from medical narratives reported at baseline, during the stay medical entrance examination. If neither ICD-10^1^ diagnosis nor textual clinical observation reported a suicide risk, we coded the predictor as “no”, versus “yes” in the opposite case | |
| Somatic comorbidity | | yes/no | yes = (a) an ICD-10^1^ diagnosis code from somatic classes^4^ was recorded between 2019 and 2022 or (b) a positive somatic disease assessment was text-mined^5^ from medical narratives reported at baseline, during the stay medical entrance examination. If neither ICD-10 diagnosis nor textual clinical observation reported a somatic comorbidity, we coded the predictor as “no”, versus “yes” in the opposite case | |
| Seclusion and mechanical restraint severity level | | Ref = 0 | No restraint was provided during the stay | |
| Ordinal variable | | 1 | A restraint was provided during the stay with (a) seclusion restraint < 168 hr and (b) physical restraint < 48 hr | |
|  | | 2 | A restraint was provided during the stay with (a) seclusion restraint ≥ 168 hr or (b) physical restraint ≥ 48 hr | |
| Hospitalization history severity level | | Ref = 0 | No hospitalization before 2019 | |
| Ordinal variable | | 1 | Number of hospitalizations before 2019 in GHU Paris psychiatry > 1 and ≤ 3 | |
|  | | 2 | Number of hospitalizations before 2019 in GHU Paris psychiatry > 3 | |
| Treatment resistance | | yes/no | yes = (a) Electroconvulsive therapy (ECT) therapy was provided during patient stay or (b) treatment resistance was text-mined^6^ during the current or previous stays. | |
| Treament interruption (due to lack of compliance) | | yes/no | yes = (a) treatment interruption event occurring during the first two days of the stay was text mined^7^ from medical narratives or (b) non-compliance to treatment during the current or previous stays was text mined from medical narratives | |

Notes:

^1^ ICD-10: International Classification of Diseases, 10th revision

^2^ data source INSERM (Institut National de la santé et de la recherche médicale)

^3^ To create lexical dictionary associated to the concepts “suicide”, we preliminary characterized the semantic field of this medical concept. To do so, we (a) reviewed ICD-10 definitions and (b) manually explored the medical narrative reports.

^4^ ICD-10 classes A00-B99, C00-D48, D50-D89, E00-E90, G00-G99, H00-H59, H60-H95, I00-I99, J00-J99, K00-K93, L00-L99, M00-M99, N00-N99, S00-T98

^5^ To create lexical dictionary associated to the concept “somatic comorbity”, we preliminary characterized the semantic field of this medical concept. To do so, we (a) reviewed ICD-10 definitions, (b) performed a manual exploration of the medical narrative reports, and (c) explored with KMEANS clustering tool the medical concepts expressed in somatic history narratives.

^6^ For psychotic disorders, we used a treatment resistance proxy based on the mention of prescription for clozapine or Leponex® (commercial name for clozapine in France) in the medical narratives. For mood disorders, we used a treatment resistance proxy based on the mention of prescription for esketamine in the medical narratives.

1. We used VIDAL drug information database to list relevant treatments for psychotic and mood disorders.
2. **Steps for selecting the most adequate statistical model**

We explored different multivariate models before selecting logistic regression for the current study. We defined how we checked model goodness and adequacy, and finally selected best model.

1. **Linear regression**

- The literature (1) (2) (3) (4) (5) encouraged us to first conduct a multivariate analysis using length of hospital stay (LOS; days) as a continuous dependant variable. Because of the skewed distribution for LOS, we first used logarithmic transformation as a link function and assumed a normal distribution for log-transformed LOS.
- We considered two possible levels for multivariate analysis: a stay level (because mean number of stays per patient, 1.5, was low) and psychiatric sector level. To check the need to account for a potential nested data structure at the psychiatric sector level (6), we ran a multilevel null model without any explanatory variables and computed the intraclass correlation coefficient (ICC). The multilevel null model without any explanatory variables gave an ICC of 6.6%. Thus, 6.6% of the total variation in the LOS was related to inter-sector differences (potentially related to environmental characteristics or sector practises). This low ICC value (<7%) did not confirm the need to account for a potential nested structure of data due to sector.
- We evaluated assumptions for linear regression by residuals analysis. We implemented the following:
  - Jarque Bera test: residuals normality hypothesis was rejected (p<0.001).
  - Breusch Pagan test: residuals homoskedasticity hypothesis was rejected (p<0.001).
  - Durbin Watson test: residuals independence hypothesis was rejected (p<0.001).

We concluded that our regression model did not verify linear assumptions. This model was not suitable for our study.

1. **Quasi Poisson model/negative binomial regression**

We successively implemented these two models (7) (8) (9) to deal with LOS overdispersion (variance/mean = 104.3). We applied the deviance test to check whether the models fitted the data correctly.

- Quasi Poisson model: model adequacy hypothesis was rejected (p < 0.001)
- Negative binomial regression: model adequacy hypothesis was rejected (p<0.001)

We concluded that these two models were not suitable for our study.

1. **Logistic model**

We finally performed logistic regression (10). To evaluate whether our final model fit the data, we used the following:

- Deviance test: p=0.7 indicated that the model fit was correct.
- Hosmer et Lemeshow test: p=0.2 indicated that the model fit was correct.
- Area under the receiver operating characteristic (ROC) curve (AUC) metric: ROC analysis demonstrated that the AUC for our final model was 65.9% (95%CI 56.3-74.9). We used a cross-validation method to avoid an over-optimistic value for AUC.

We leveraged the variance inflation factor (11) to detect multicollinearity between variables. Adjusted odds ratios (ORs) were computed with their confidence intervals (CIs). We used InteractionR package (R v4.3.1) (12) to compute the 95% CI for the somatic comorbidity OR in female sex strata.

**Conclusion**

Statistical tests showed that neither linear regression nor quasi-Poisson or non-negative binomial regression was suitable to explain the LOS outcome for our dataset. We selected a logistic regression model, which seemed the most suitable for our data and study objective.

1. **Complementary information about missing data handling**
2. We compared groups: excluded stays with missing data (n=2,664) versus observations with no missing data (n=6,206). Hence, we ran a bivariate analysis for the variables sex, age, and LOS using (a) the chi-squared test for sex and (b) Wilcoxon rank test for the continuous variables age and LOS (variables with non-normal distribution in each sample, according to the Shapiro-Wilk test of normality). Observations with missing values excluded from the database (n=2,664) were significantly associated with male sex (p=0.01), older age (p<0.001), and long LOS (p<0.001) as compared with observations included in the dataset (n=6,206).
3. Multivariate analysis run on imputed data (n=8,870) showed consistent results with the primary logistic regression, run on a dataset of 6,206 observations. It also revealed diagnosis organic mental disorders as a risk factor (OR = 1.6 [95%CI 1.1-2.2]).
4. We ran multivariate analysis on a dataset (n=8,522 stays) from which marital status was removed (this feature accounted for most of the missing data). Results were consistent with the primary logistic regression, run on a dataset of 6,206 observations.
5. **Complementary information about sensitivity analysis of cut-off values for binomial logistic regression**

Sensitivity analysis was conducted with different LOS cut-off values for, successively 20, 60, 90 days. To summarize, we found globally consistent results with the 30-day cut-off value. Still some new significant associations were revealed and others disappeared:

- **For cut-off = 20 days**: new associations with the LOS outcome were diagnoses such as mental and behavioral disorders related to alcohol use (OR = 1.2 [95%CI 1.1-1.4]) and organic mental disorders (OR = 1.7 [1.1-2.5]). Treatment resistance was no longer significantly associated with long LOS.
- **For cut-off = 60 days**: a new association with the LOS outcome was organic mental disorders (OR = 1.8 [1.1-2.9]). The interaction between sex and somatic comorbidity was no longer significantly associated with long LOS, nor was admission from outside the sectorization zone.
- **For cut-off = 90 days**: new associations with the LOS outcome were organic mental disorders (OR = 2.8 [1.6-4.6]) and homelessness (OR = 1.9 [1.3-2.7]). The interaction between sex and somatic comorbidity was no longer significantly associated with long LOS, nor was admission from outside the sectorization zone, being in a pathological journey, or adult personality and behavior disorders. However, we found a significant interaction between sex and pervasive and specific developmental disorders.

**References:**

1. Carranza Navarro F, Álvarez Villalobos NA, Contreras Muñoz AM, Guerrero Medrano AF, Tamayo Rodríguez NS, Saucedo Uribe E. Predictors of the length of stay of psychiatric inpatients: protocol for a systematic review and meta-analysis. Syst Rev. 2 mars 2021;10(1):65.

2. Baeza FL, da Rocha NS, Fleck MP. Predictors of length of stay in an acute psychiatric inpatient facility in a general hospital: a prospective study. Braz J Psychiatry. 2018;40(1):89‑96.

3. Dimitri G, Giacco D, Bauer M, Bird VJ, Greenberg L, Lasalvia A, et al. Predictors of length of stay in psychiatric inpatient units: Does their effect vary across countries? Eur Psychiatry. févr 2018;48:6‑12.

4. Gopalakrishna G, Ithman M, Malwitz K. Predictors of length of stay in a psychiatric hospital. Int J Psychiatry Clin Pract. 2015;19(4):238‑44.

5. Tulloch AD, Fearon P, David AS. Length of stay of general psychiatric inpatients in the United States: systematic review. Adm Policy Ment Health. mai 2011;38(3):155‑68.

6. Gandré et al. Coralie Gandré, Jeanne Gervaix, Julien Thillard, Jean-Marc Macé, Jean-Luc Roelandt, et al. The Development of Psychiatric Services Providing an Alternative to Full-Time Hospitalization Is Associated with Shorter Length of Stay in French Public Psychiatry. International Journal of Environmental Research and Public Health.

7. Jacobs R, Gutacker N, Mason A, Goddard M, Gravelle H, Kendrick T, et al. Determinants of hospital length of stay for people with serious mental illness in England and implications for payment systems: a regression analysis. BMC Health Services Research. 30 sept 2015;15(1):439.

8. Baum F. Lengths of inpatient stay and sick leave of patients with mental diseases: disorder-specific effects of flexible and integrated treatment programs in Germany.

9. Wolff J, McCrone P, Patel A, Kaier K, Normann C. Predictors of length of stay in psychiatry: analyses of electronic medical records. BMC Psychiatry. 7 oct 2015;15(1):238.

10. Cheng P, Wang L, Xu L, Zhou Y, Zhang L, Li W. Factors Related to the Length of Stay for Patients With Schizophrenia: A Retrospective Study. Frontiers in Psychiatry [Internet]. 2022 [cité 2 janv 2024];12. Disponible sur: https://www.frontiersin.org/articles/10.3389/fpsyt.2021.818254

11. O’brien RM. A Caution Regarding Rules of Thumb for Variance Inflation Factors. Qual Quant. 11 sept 2007;41(5):673‑90.

12. Alli BY. InteractionR: An R package for full reporting of effect modification and interaction. Software Impacts. 1 nov 2021;10:100147.
